# Microbial Interaction is Among the Key Factors for Isolation of Previous Uncultured Microbes

**DOI:** 10.1007/s12275-023-00063-3

**Published:** 2023-08-17

**Authors:** Chang Yan, Jeffrey S. Owen, Eun-Young Seo, Dawoon Jung, Shan He

**Affiliations:** 1 Li Dak Sum Yip Yio Chin Kenneth Li Marine Biopharmaceutical Research Center, College of Food and Pharmaceutical Sciences, Ningbo University, Ningbo, 315832 People’s Republic of China; 2 Ningbo Institute of Marine Medicine, Peking University, Ningbo, 315832 People’s Republic of China; 3 Department of Environmental Science, Hankuk University of Foreign Studies, Yongin, 17035 Republic of Korea

**Keywords:** Uncultured microbes, Microbial uncultivability, Microbial cultivation, Growth factors

## Abstract

Pure cultivation of microbes is still limited by the challenges of microbial uncultivability, with most microbial strains unable to be cultivated under standard laboratory conditions. The experience accumulated from advanced techniques such as in situ cultivation has identified that microbial interactions exist in natural habitats but are absent in laboratory cultures. These microbial interactions are likely one of the key factors in isolating previously uncultured microbes. The need for better knowledge of the mechanisms operating in microbial interactions has led to various experiments that have utilized microbial interactions in different approaches to microbial cultivation. These new attempts to understand microbial interactions not only present a new perspective on microbial uncultivability but also provide an opportunity to access uncultured phylogenetically novel microbes with their potential biotechnology applications. In this review, we focus on studies of the mechanisms of microbial interaction where the growth of other microbes is affected. Additionally, we review some successful applications of microbial interactions in cultivation methods, an approach that can play an important role in the bioprospecting of untapped microbial resources.

## Introduction

It is widely known that cultured microbes represent a small fraction of all microbial diversity on our planet. Only relatively few microbes can grow into colonies on standard agar media, thus leading to the ‘great plate count anomaly,’ a recognized scientific challenge for over 120 years (Amann et al., 1995; Staley & Konopka, 1985). This most fundamental unsolved problem in microbiology still exists as a major bottleneck in the field of biological exploration of microbial resources.

There are various assumptions and hypotheses for microbial uncultivability in the laboratory, including a lack of essential nutrients or other conditions present in incubation techniques (Stewart, 2012). However, no studies have led to a full resolution of the phenomenon of microbial uncultivability. This suggests that microbial uncultivability cannot simply be explained by the unfitness of specific strains to certain culture conditions such as media composition, gas composition, temperature, or pH (Jung et al., 2021a). More complicated and unknown factors that critically affect the growth of such uncultured microbes are absent from the standard techniques for microbial cultivation.

In nature, microbes do not exist as individuals but engage in complex ecological interactions (Faust & Raes, 2012; Haruta & Kanno, 2015; Niepa et al., 2016; Zengler & Zaramela, 2018). Therefore, the microbes within ecological networks often rely on neighboring organisms to acquire signaling molecules, which help them to survive and propagate in nature (D’Onofrio et al., 2010). Namely, microbial interaction contributes to facilitating the growth of other community members in the natural environment (Nichols et al., 2008). Thus, recent studies have pointed out that the microbial interactions present in natural environments but absent in laboratory culture conditions are one of the important factors in regulating microbial growth.

However, observing interactions in microbial communities is a huge challenge because of their extraordinary complexity, unpredictability (Faust & Raes, 2012), and the limitations of techniques to observe interactions under conditions that are similar to natural conditions (Zelezniak et al., 2015). Thus, it is generally agreed that our knowledge of microbial interactions in microbial communities and their applications in cultivation strategies have not been fully examined.

In this review, we introduce the current limitations of microbial cultivation and some proposed applications of microbial interactions as key factors to overcome the limitations in microbial cultivability. We summarize studies that have led us to understand that microbial interactions are important for the growth of some previously uncultured microbes and discuss the potential for utilizing microbial interactions in approaches to laboratory microbial cultivation.

## Clue to Access ‘Uncultures’ from in situ Cultivation Studies

Although there are arguments over the proportion of uncultivated microbes in nature (Martiny, 2020; Martiny et al., 2019; Steen et al., 2019), culture-independent surveys have clearly indicated that the high diversity of unknown microbes, the so-called “microbial dark matter,” are numerically dominant in all major environments on our planet (Handelsman, 2004; Locey & Lennon, 2016). It would be reasonable to think that differences between the laboratory environment where microbes are cultivated and the environment where they exist in nature are the main cause of microbial uncultivability. However, few studies have identified the factors that decisively explain microbial uncultivability.

One simple solution to the problem of unidentified growth factors is to cultivate the microbes in their natural environments. Applying this idea to laboratory microbial cultivation led to the development of various in situ cultivation methods (e.g., diffusion chamber and ichip) aiming to better simulate the natural environment (Jung et al., 2021b). Several in situ cultivation approaches that share the same basic rationale have been tested in diverse environments, e.g., activated sludge, alkaline soda lakes, hot springs, sediments, sponges, and soil environments. These have led to the isolation of many previously uncultured microbes (Bollmann et al., 2007; Jung et al., 2013, 2014, 2016, 2018; Steinert et al., 2014).

**Fig. 1:**
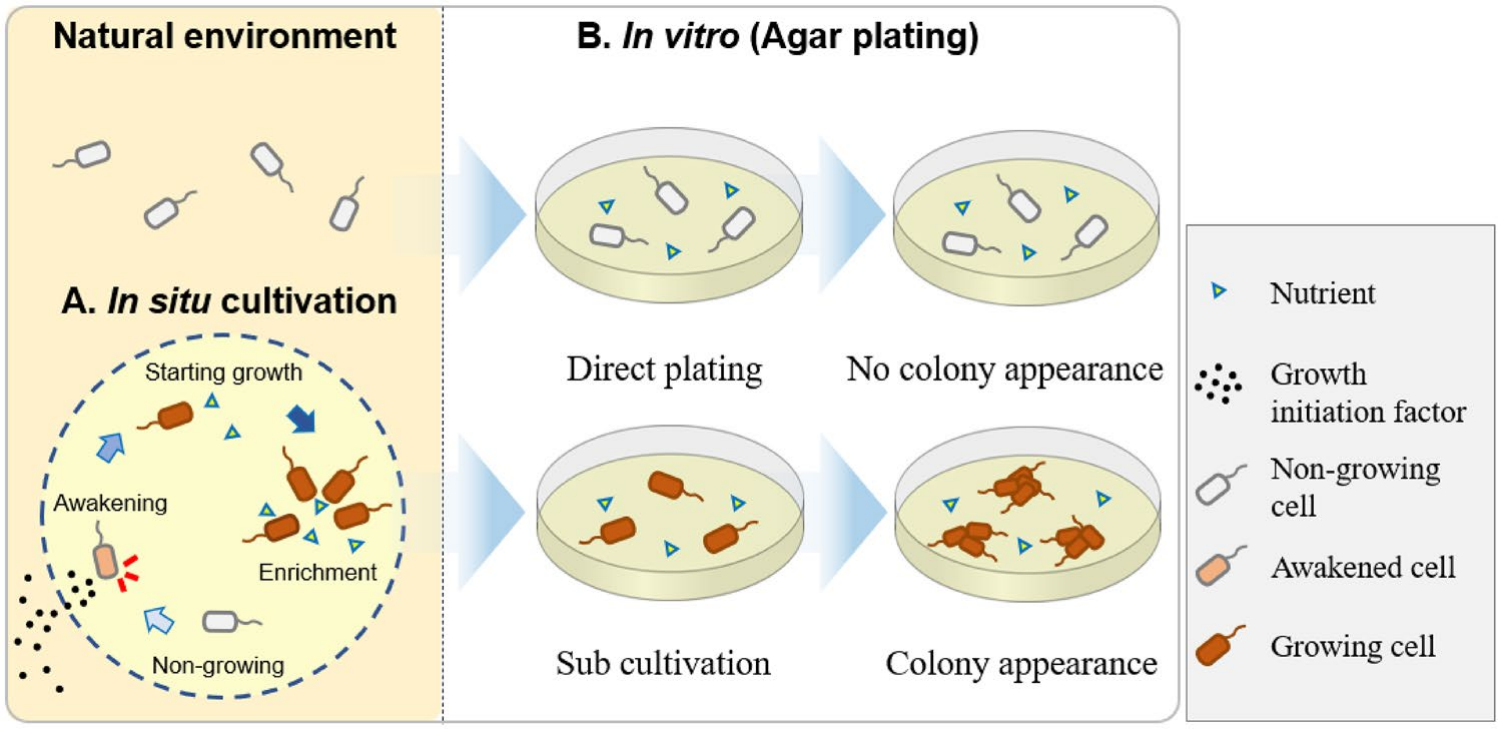
Hypothesis explaining how in situ cultivation cultivates previously uncultured bacteria. During in situ incubation, microbes are induced from a non-growing state by resuscitation factors from the natural environment, then regrow and become enriched, supported by nutrients in the chamber (**A**). Most microbes from nature do not recover from a non-growing state without resuscitation factors and thus do not form colonies, while some microbes were sub-cultured after the in situ cultivation and maintain their growth agar plates (**B**) (Jung et al., 2021b)

Recently, Jung et al. (2021b) clarified one key mechanism for in situ cultivation in a marine sponge where a growth initiation factor in the natural environment (sponge extract in that study) stimulated bacterial resuscitation from a nongrowing state. Their results suggest two important points. First, during in situ incubation, microbes are induced from a non-growing state by unknown growth factors from the environment (Fig. 1A). Second, after in situ cultivation, grown strains maintain their growth on standard agar plates during sub-cultivation, resulting in a visible colony appearance (Fig. 1B). Therefore, during the in situ cultivation, “resuscitation factors” were provided from the natural environment (i.e., the marine sponge), while nutrients were provided inside the chamber (in situ cultivation device; diffusion chamber or ichip) from the beginning. These unknown factors enabled some microbes to begin growing and finally become enriched inside the chamber (Fig. 1A). Since sponge-associated microbes produce various chemical compounds inside their host, the unknown resuscitation factors are likely also produced by specific microbes associated with the sponge (Wang, 2006), an example of microbial interaction. Therefore, we expect microbial interactions to be among the key factors to access some previously uncultured microbes.

## Observation of Microbial Interactions in Nature

Early methods for predicting microbial interactions in the environment used abundance data for the microbial community (Faust & Raes, 2012). This approach is straightforward; when two species (or any taxonomically relevant units) co-occur or show a similar abundance pattern over multiple samples, a positive relationship is assumed; when they show mutual exclusion or anticorrelation, a negative one is inferred. Culture-independent molecular tools such as metagenomic and 16S rRNA pyrosequencing analysis have accelerated this analysis by helping to observe microbial communities and their dynamics in microbial ecosystems using their 16S rRNA gene sequences (Nurul et al., 2019). Any indicated symbiosis and related patterns found in these datasets can be used to predict species interactions in a variety of environments. Therefore, analyses that have identified microbial interactions have opened the way to the discovery of cooperative and competitive relationships between species (Faust & Raes, 2012). Related studies have suggested that environmental microbial interactions are key strategies to survive in different environments and may maintain diversity in the microbial community. For example, interactions occur through the transmission of molecular and genetic information, and these interactions involve a variety of mechanisms, such as secondary metabolites, siderophores, quorum sensing systems, and others. Dimitriu et al. (2014) proposed an experimental framework that linked the exchange of genetic information to the selection of cooperative traits. They demonstrated that horizontal gene transfer can favor interactions by controlling the secretion of shared molecules and plasmid conjugations using simulations based of synthetic bacterial systems. Braga et al. (2016) claimed that the ultimate unit of interaction is the gene expression of an organism in response to an environmental stimulus, which is responsible for producing the molecules involved in the interactions. With a better understanding of microbial interactions, can we improve the isolation efficiency of previously uncultured microbes? Combining our understanding of microbial interactions with new microbial culture techniques can make this possible.

## Microbial Cultivation Using Microbial Interactions

### Promoting the Growth of Specific Microbial Species Using Microbial Interactions

**Fig. 2:**
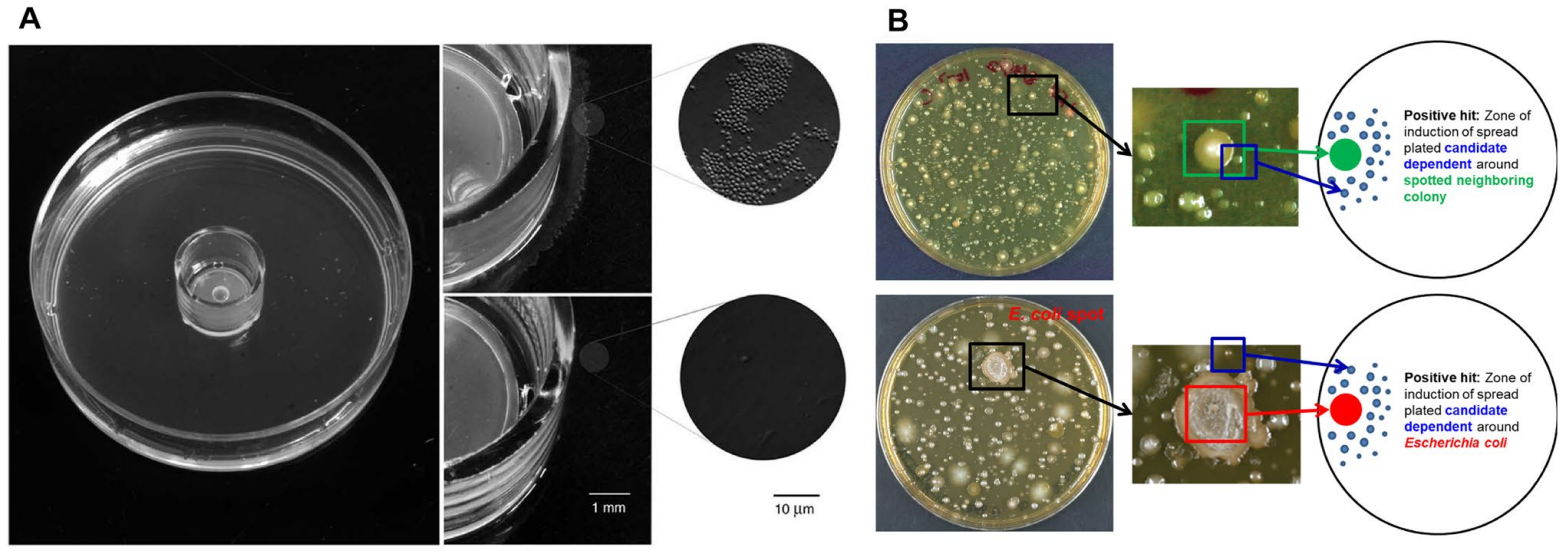
Simple attempts at utilizing microbial interactions. **A** Growth induction by helper strain. The two microbes were co-cultured on the same plate, and the results showed that the microcolony count was significantly higher in the presence of helper bacteria (Nichols et al., 2008). **B** Isolating uncultured bacteria using a co-culture method. The results for both targeted and untargeted methods showed a positive interaction for dependent bacteria around the helper bacteria (D’Onofrio et al., 2010)

Studies applying microbial interactions to cultivation studies are summarized in Table 1. As mentioned, early studies focused on the apparent influence of one strain on the growth of another strain. Nichols et al. (2008) isolated previously uncultured bacteria by in situ cultivation using a diffusion chamber and attempted to identify the mechanism of interspecific microbial interactions. They performed co-cultivation in a pairwise manner among the isolates from the diffusion chambers (Fig. 2A). The results showed that one *Cellulophaga lytica* could induce the growth of previously uncultured *Psychrobacter* sp. when those isolates grow together, indicating that *Psychrobacter* sp. can grow on a normal medium in the laboratory with the ‘helper’ strain. Their findings suggested that at least some uncultured microbes could grow on a standard medium if paired with another species from the same environment. Through the isolation and identification of the growth factors for the helper strain, they found one 5-amino-acid peptide that was able to induce previously uncultured *Psychrobacter* sp. to grow on a standard agar medium.

**Table 1:** Examples of studies that included microbial interactions in cultivation studies, and some characteristics of each study

Technique	Key factor	Target microbes	References
Promoting the growth of microbial species using microbial interactions	Activity of a short peptide induces growth of previously uncultured strains	Marine microbes	Nichols et al. (2008)
	Siderophores induce growth of uncultured marine bacteria		D’Onofrio et al. (2010)
	Quinones can be growth factor for human gut microbiota	Human gut microbiome	Fenn et al. (2017)
	Anaerobic co-culture can change the metabolic network for growth	*G. sulfurreducens* and *S. oneidensis*	Kane et al. (2021)
Microbial interactions in microbial networks	Co-culture promotes positive interaction for the growth of non-growing strain	Soil bacteria	Kehe et al. (2021)
	Spent-culture medium (SCM) can effectively promote growth of previously uncultivated bacteria	Co-occurrence networks in a hot spring	Xian et al. (2020)

Studies listed in the table follow the order discussed in the text

Another similar study by the same research group (D’Onofrio et al., 2010) showed that small molecular iron chelators and siderophores can induce the growth of previously uncultured marine bacteria. They also conducted co-culture methods on selected strains isolated from marine sediment samples using a diffusion chamber to detect factors that could induce the growth of previously uncultured marine bacteria. This indicates that siderophores from the neighboring helper strain are able to induce the growth of some previously uncultured marine bacteria (Fig. 2B). Their findings showed that because those strains (previously uncultured bacteria) cannot produce siderophores autonomously, they are chemically dependent on other members (helper stains) in the natural environment.

Later, Fenn et al. (2017) explored whether a similar phenomenon occurs in the human gut microbial ecosystem. They performed co-culture experiments with microbes from human gut flora and identified quinones as a new growth factor that can induce the growth of other bacteria. The result showed that eight induced strains formed a growth gradient around the culturable helper strains, suggesting that some slow-growing bacteria were dependent on the faster-growing bacteria nearby. While multiple helper strains were identified, they found that *Escherichia coli* was also capable of promoting the growth of all induced isolates. Screening a knockout library of *E. coli* showed that a menaquinone biosynthesis pathway was required to induce the growth of the induced isolates. Therefore, their study concluded that quinones can be used to improve existing bacterial growth media or modulate human gut microbiota by encouraging the growth of important symbionts.

In studies with a similar focus, Kane et al. (2021) used an anaerobic co-culture system with *Shewanella oneidensis* and *Geobacter sulfurreducens*, two previously non-interacting bacteria, to understand microbial interactions in a more complex setting, and to investigate how anaerobic microbial communities might form. Through the use of synthetic biology, they analyzed a co-culture system in which a facultative anaerobe (*S. oneidensis*) consumes glycerol and supplies acetate to a strict anaerobe (*G. sulfurreducens*). Under the experimental conditions, the growth of *G. sulfurreducens* was dependent on the presence of *S. oneidensis*, indicating that the metabolic pathway utilized in the co-culture was essential for inter-species interaction and that the bacterial metabolites released during growth likely contain signaling compounds that regulate microbial communication.

### Application of Microbial Interactions in Microbial Networks

**Fig. 3:**
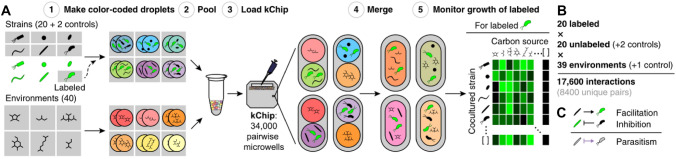
High-throughput interaction device for microbial cultivation. **A** Bacteria from different carbon sources were mixed with labeled strains on a kChip to form numerous co-cultures. KChip produces co-cultures at an ultra-high throughput scale by rapidly and randomly combining the components of the medium in droplets and/or micropores containing microbial cultures. **B** Overall size of the kChip screen. **C** Using data across kChips, bidirectional interactions were inferred by combining data where each strain within a given pair was the labeled strain (Kehe et al., 2021)

Based on the results of studies that investigated the growth of specific microbial strains using microbial interactions, new attempts are being made to apply the same basic rationale to unspecified multiple microbial species. Kehe et al. (2021) designed the kchip, an ultra-high-throughput co-culture platform to measure complex interactions between bacteria in different carbon source conditions. In that study, 180,408 interactions were measured between 20 soil bacteria in 40 carbon environments (Fig. 3). The results indicate that positive interactions usually occur as parasitic relationships in strains with different carbon consumption statuses, and the difference in carbon sources also affects the prevalence of positive interactions. Notably, positive interactions (usually in the form of parasitism) often occurred between strong and weak growers, but also sometimes between weak growers, regardless of the carbon source or strain pair. The findings from that study offer two key lessons. First, microbial interactions can occur in various forms depending on environmental conditions, rather than simply appearing between each specific species. This suggests that such interactions do not occur one-to-one but as multiple interactions depending on the complex effects among different species in various environments. Second, microbial interactions occur extensively and frequently among microbes and significantly impact the microbial community's species compositions. Standard cultivation in the laboratory cannot reflect these microbial interactions, and therefore interactions are among the key factors that contribute to the difference in microbial composition between natural environments and cultured isolates.

Based on this idea, incorporating microbial interactions into laboratory or *in-situ* microbial cultivation experiments should be considered. Xian et al. (2020) targeted isolating the previously uncultured *Chloroflexus* group in hot spring environments with a focus on microbial interactions. First, they found that, in the conditions found in a hot spring environment, species with low abundance (e.g., *Tepidimonas.* sp., less than 1%) have high centrality and were defined as ‘key nodes’, while the most abundant species was *Chloroflexus* sp. (13.9%) according to microbial co-occurrence networks analysis. Then, they hypothesized that the highly centralized key node bacteria in hot spring microbial communities, such as *Tepidimonas*, can facilitate neighboring microbes to play a role in organizing and regulating the community. Therefore, they chose the key node bacteria as a growth-promoting species to target and isolate some previously uncultivated strains of the phylum *Chloroflexi* that are abundant in nature but rarely grow in the laboratory. A growth-promoting experiment was conducted to confirm the efficiency of the key node bacteria, and it was determined that *Tepidimonas* sp. can promote the growth of *Chloroflexus* sp. They added 10% *Tepidimonas* supernatant to the medium and carried out targeted isolation of *Chloroflexus* sp. from the natural environment. Their results showed that most of the isolated *Chloroflexus* sp. were previously uncultured. Metabolite studies have also shown that the supernatant from liquid culture contains some low-molecular-weight organic compounds and has the potential to promote the growth of these bacteria (Xian et al., 2020).

There are other studies that indicate microbial interaction can be one of the key factors for isolating previous uncultured microbes, although the interaction was not a major topic in that research. Kim et al. (2021) described heme deficiency in a rich lineage of aquatic microbes. They demonstrated that the acI lineage, a dominant free-living bacterial population in freshwater habitats, is dependent on external sources of heme. Their study found that all isolated acI strains lacked essential enzymes necessary for heme biosynthesis, suggesting that heme auxotrophy was a conserved feature of this lineage. Analyses of > 24,000 representative genomes for species clusters of the Genome Taxonomy Database showed that heme auxotrophy is widespread across abundant but previously uncultured microbes. Therefore, heme auxotrophy is a more common phenomenon than conventional thought, and their findings may lead to use of heme as a growth factor to improve the cultured microbial diversity.

Imachi et al. (2020) designed and operated a methane-fed continuous-flow bioreactor system. They enriched anaerobic microbes from marine sediment in the reactor for more than 2000 days and they successfully cultured important microbial groups such as Asgard archaea, that are known to have a wide range of physiological properties, but were previously uncultured. In their system, specific target bacteria were able to grow synthetically with other *Methanobacterium* sp. Their work showed that microbial interactions in the system was one of the factors that facilitated the cultivation of the target species. Thus, interactions between microbial strains can be utilized in a variety of microbial cultivation techniques, likely for a large number of unidentified microbial species from natural environments.

### Cultivation Using Control of Competitive or Negative Interactions

In contrast, some microbial species would be expected to have a negative effect on the growth of other species, resulting in culture collections with skewed species distributions. For example, when multiple species share the same resources, the winner is always the faster growers in laboratory culture condition, even if the slower species (most likely uncultivated) is the dominant type in the environment (Jung et al., 2014, 2018).

To avoid this distortion, some studies have attempted to reduce or avoid such negative effects. Davis et al. (2005) used diluted nutrient broth medium (1% of the manufacturer’s suggested concentration) to isolate bacteria from soil samples, and they extended the incubation time to 3 months. Many strains that were previously uncultured were isolated on low concentration agar media, suggesting that reducing the negative effects on other species can result in completely different culture results. One method using a similar principle, single cell cultivation (Connon and Giovannoni, 2002), has also proved successful for the culture of previously uncultured marine planktonic bacteria, which have been found in marine clone libraries, but not in culture collection. In this study, very-low-nutrient media was used to limit fast growers, and the number of microbes isolated using nutrient limited media was 14 to 1400-fold higher than that of traditional microbial culturing methods.

Some studies have used this negative interaction to cultivate targeted microbes. The marine sponge, *Theonella swinhoei*, is an arsenic hyper-accumulator species, harboring large number of associated bacteria. Keren et al. (2016) isolated arsenic-tolerant bacteria associated with sponges by adding arsenic to the culture media and classified them into 15 operational taxonomic units (OTUs). This suggests that some negative interactions can be utilized to target the isolation of specific microbes (arsenic-tolerant bacteria in that study), while at the same time risking the loss of more novel non-target strains and disrupting the diversity of microbial communities.

## Future Directions in Microbial Cultivation Using Microbial Interactions

Several studies reviewed here clearly suggest that microbial interactions are among the main factors that affect microbial diversity in microbial communities. The application of these interactions, which have not been fully reflected in standard cultivation techniques to date, should lead to the isolation of previously uncultivable microbes.

However, this new challenge still faces difficulties and limitations. Despite its importance, the methods for observing and measuring microbial interactions among microbes are not yet standardized due to their complexity and unpredictability (Faust & Raes, 2012) as well as the limitations of techniques to observe microbial interactions in more natural conditions (Zelezniak et al., 2015). The development of new techniques to understand microbial interactions in a diverse range of microbial communities is needed. This might include the application of molecular biological approaches. In addition, understanding the mechanisms for microbial interaction should be prioritized in studies on microbial cultivation. For example, because isolating cells is merely an initial step, more research is needed to elucidate the exact mechanisms of interaction. Furthermore, to continue moving forward on experiments in microbial cultivation, physiological analysis using pure isolates of strains that have been shown to affect the growth of other microbial strains should be emphasized, and purification and identification of compounds identified as facilitating interactions should be another focus of research efforts. The diversity and specificity of interactions between different microbes, as well as their mechanism, should also be taken into account.

In our review, we primarily focus on the effects of metabolites and compounds secreted by microbes as related to microbial cultivation. However, there are other factors that can have a role in interactions among microbial species. For example, physical contact, physicochemical conditions, or the influence of solid or liquid environments are also factors that might affect microbial interactions.

In summary, incorporating microbial interactions in microbial cultivation experiments is certainly needed to push forward the somewhat traditional techniques still used in microbial cultivation. Even though the mechanisms for microbial interactions are not yet well understood and only a few studies have identified specific metabolic compounds involved in microbial interactions. We expect that novel techniques that incorporate microbial interactions in cultivation experiments will play an important role in continuing the exploration of undiscovered microbial resources.

## Data Availability

The data that support the findings of this study are available from the corresponding authors upon reasonable request.
